# Pristimerin Promotes Ubiquitination of HSPA8 and Activates the VAV1/ERK Pathway to Suppress TNBC Proliferation

**DOI:** 10.1002/advs.202413174

**Published:** 2025-01-15

**Authors:** Qin‐Wen Liu, Qi‐Ling Fan, Jia‐Ying Chen, Jing‐Xin Liu, Yi Li, Qian Luo, Yu‐Peng Chen, Hang‐Tian Wu, An‐Qi Xu, Sheng Wang, Ai‐Ping Lu, Dao‐Gang Guan

**Affiliations:** ^1^ Neurosurgery Center Department of Cerebrovascular Surgery Engineering Technology Research Center of Education Ministry of China on Diagnosis and Treatment of Cerebrovascular Disease Zhujiang Hospital Southern Medical University Guangzhou 510282 P. R. China; ^2^ Department of Biochemistry and Molecular Biology School of Basic Medical Sciences Southern Medical University Guangzhou 510080 P. R. China; ^3^ School of Chinese Medicine Hong Kong Baptist University Hong Kong SAR 999077 P. R. China; ^4^ Guangdong Cardiovascular Institute Guangdong Provincial People's Hospital Guangdong Academy of Medical Sciences Guangzhou Guangdong 510000 P. R. China; ^5^ Institute of Integrated Bioinformedicine and Translational Science Hong Kong Baptist University Hong Kong SAR 999077 P. R. China; ^6^ Division of Orthopaedics and Traumatology Department of Orthopaedics Nanfang Hospital Southern Medical University Guangzhou Guangdong 510515 P. R. China; ^7^ Beijing Anzhen Hospital Capital Medical University Beijing 100029 P. R. China; ^8^ Guangdong‐Hong Kong‐Macau Joint Lab on Chinese Medicine and Immune Disease Research Guangzhou Guangdong 510000 P. R. China

**Keywords:** HSPA8, pristimerin, triple‐negative breast cancer, VAV1/ERK pathway

## Abstract

Triple‐negative breast cancer (TNBC) is a subtype of breast cancer with a poor prognosis. The natural compound pristimerin has shown promising anti‐tumor effect. Here, it is found that pristimerin significantly triggered the activation of autophagy initiation and induced apoptosis in TNBC. Mechanistically, RNA sequencing revealed that pristimerin activated mitogen‐activated protein kinase/extracelluar regulated protein kinases (MAPK/ERK) pathway. Drug affinity responsive target stability and mass spectrometry techniques are employed to confirm the direct binding target of pristimerin. Heat shock protein family A member 8 (HSPA8) is identified and verified by cellular thermal shift assays and surface plasmon resonance assays. The further results suggested that pristimerin promoted the ubiquitination and degradation of HSPA8, leading to a decrease in the degradation of Vac Guanine Nucleotide Exchange Factor 1 (VAV1), a downstream client protein of HSPA8 which plays a crucial role in activating the ERK pathway. Importantly, knockdown of HSPA8 or VAV1 significantly impaired the anticancer activity of pristimerin on TNBC cells. Additionally, pristimerin significantly inhibited the migration and invasion of TNBC cells and enhanced the sensitivity of TNBC cells to doxorubicin. Collectively, this study provides the initial evidence that pristimerin directly targets HSPA8 to activate the VAV1/ERK pathway, thereby promoting cell autophagy and apoptosis.

## Graphical abstract

Triple‐negative breast cancer has a poor prognosis. Pristimerin shows anti‐tumor effects by activating autophagy and apoptosis. It directly targets HSPA8, leading to VAV1 degradation and activation of the MAPK/ERK pathway. In addition, it enhances TNBC cell sensitivity to doxorubicin and inhibits cell migration and invasion, suggesting its potential as a therapeutic agent against TNBC.

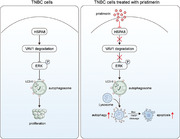

## 1 Introduction

Triple‐negative breast cancer (TNBC) is defined by the absence of estrogen receptor and progesterone receptor expression, as well as the absence of human epidermal growth factor receptor 2 overexpression or gene amplification. This particular subtype of breast cancer (BCa) is notorious for its high mortality rate, primarily attributed to its significant heterogeneity, aggressive behavior, and scarcity of viable therapeutic targets. Presently, chemotherapy remains the primary treatment option for TNBC^[^ ^1^ ^]^; however, patients frequently develop resistance to these drugs.^[^ ^2^ ^]^ Consequently, the pursuit of more efficacious and safer anticancer medications to eradicate tumors has become a shared objective within the medical community.

Traditional Chinese medicine (TCM) has been utilized for centuries as an effective treatment for cancer due to its minimal side effects and high efficacy.^[^ ^3^ ^]^ Extensive evidence supports the anticancer properties of various natural products derived from TCM, including resveratrol,^[^ ^4^ ^]^ apigenin,^[^ ^5^ ^]^ luteolin,^[^ ^6^ ^]^ curcumin^[^ ^7^ ^]^, and pristimerin.^[^ ^8^ ^]^ Among these compounds, pristimerin, a naturally occurring quinone methide triterpenoid from the Tennessee and Hippocampus families,^[^ ^9^ ^]^ has demonstrated a wide range of biological effects, such as anti‐inflammatory,^[^ ^10^ ^]^ antibacterial,^[^ ^11^ ^]^ antifungal,^[^ ^12^ ^]^ and antitumor^[^ ^13^ ^]^ activities. Notably, recent studies have unveiled the potential of pristimerin as an anticancer agent against various types of tumors. For instance, pristimerin exhibits inhibitory effects on colorectal cancer by suppressing the inflammatory response and the Wnt/β‐catenin pathway.^[^ ^14^ ^]^ In addition, it can overcome drug resistance in BCa cells by inhibiting the protein kinases B pathway,^[^ ^15^ ^]^ and induce apoptosis and autophagy in esophageal cancer cells.^[^ ^16^ ^]^ Nevertheless, the precise function and underlying mechanism of action of pristimerin in TNBC remain largely unexplored.

Heat shock protein family A member 8 (HSPA8), a member of the HSP70 family, plays a crucial role in maintaining cellular homeostasis. It is the only chaperone protein known to facilitate chaperone mediated autophagy (CMA), a cellular recycling program. Numerous studies have demonstrated that the expression of HSPA8 is upregulated in various cancer cells. For instance, in chronic granulocytic leukemia, targeting HSPA8 to downregulate BCR‐ABL not only inhibited cancer cell proliferation but also increased cancer cell sensitivity to imatinib.^[^ ^17^ ^]^ In colon cancer, HSPA8 interacted with member RAS oncogene family (Rab1A) in a chaperone‐dependent manner, which prevented the degradation of Rab1A denatured by stress exposure, consequently promoting cancer cell survival under stressful conditions.^[^ ^18^ ^]^ In gliomas, HSPA8 facilitated the growth of glioma cells by interacting with the β‐1,4‐aglactosyltransferase protein.^[^ ^19^ ^]^ Overall, the inhibition of HSPA8 significantly reduced cancer cell proliferation, migration, and invasion, while promoting apoptosis.

In this study, our findings demonstrated that pristimerin effectively suppresses the proliferation, migration, and invasion of TNBC cells. To elucidate the underlying molecular mechanisms, we employed drug affinity responsive target stability‐mass spectrometry (DARTS‐MS) technology to identify the specific proteins that pristimerin binds to. Consequently, HSPA8 was proved as a direct binding target of pristimerin. By promoting the ubiquitination degradation of HSPA8, pristimerin impedes the degradation of its downstream client protein vav guanine nucleotide exchange factor 1 (VAV1). The increased level of VAV1 protein further promoted the activation of extracellular regulated protein kinases (ERK) pathway, and ultimately facilitated the occurrence of cell autophagy and apoptosis.

## 2 Results

### 2.1 Pristimerin Inhibits TNBC Cell Proliferation and Induces Apoptosis

**Figure 1:**
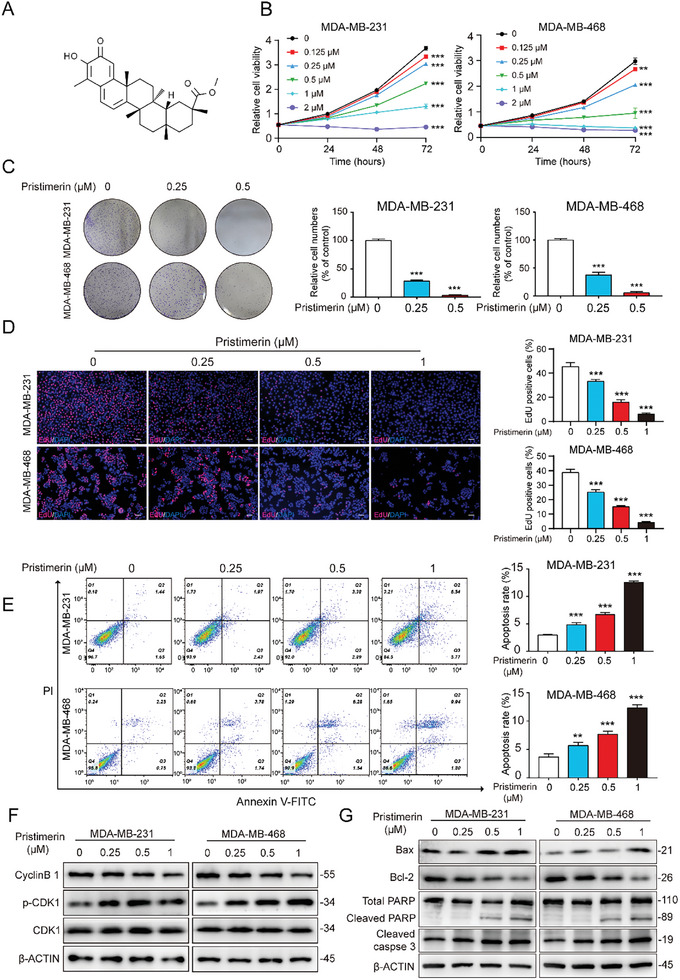
Pristimerin inhibits TNBC cell proliferation and induces apoptosis. (A) The chemical structure of pristimerin. (B) Cell viability was assessed using the CCK‐8 assay after treatment with pristimerin for indicated times. (C) The colony formation was evaluated after treatment with pristimerin. (D) The proliferation ability was detected by EdU assay. Red: EdU‐positive cells. Scale bar = 100 µm. (E) Cell apoptosis was analyzed using flow cytometry after the treatment of pristimerin for 48 h. (F) The expressions of CyclinB 1, p‐CDK1 and CDK1 were detected by Western blot after TNBC cells were treated with pristimerin for 48 h. (G) The expressions of apoptosis‐related proteins Bax, Bcl‐2, Cleaved caspase 3 and Cleaved PARP were detected by Western blot after TNBC cells were treated with pristimerin for 48 h. Bars, SDs; ^*^0.01 < *p* < 0.05, ^**^0.001 < *p* < 0.01, and ^***^ *p* < 0.001.

Pristimerin, a naturally occurring quinone methide triterpenoid, was identified as a potential inhibitor of TNBC development (**Figure** **1**A). To evaluate the impact of pristimerin on TNBC cell proliferation, various concentrations of pristimerin were administered to MDA‐MB‐231 and MDA‐MB‐468 cells for up to 5 days, and then the relative cell viability was tested through cell counting kit‐8 (CCK‐8) reagent. The results demonstrated that pristimerin effectively inhibited TNBC cell proliferation in a time‐ and concentration‐dependent manner (Figure 1B). Furthermore, the results from the colony formation assay showed a remarkable decrease in the number of TNBC cell clones following pristimerin treatment (Figure 1C). Moreover, the 5‐ethynyl‐2‐deoxyuridine (EdU) staining results revealed a gradual decrease in the number of newly synthesized DNA labeled with red fluorescent dye EdU with pristimerin treatment (Figure 1D); And the cell cycle results showed a G2/M phase arrest in TNBC cells with pristimerin (Figure S1A, Supporting Information), which was further corroborated by the progressively higher levels of phosphorylated cyclin dependent kinase 1 (p‐CDK1) expression and progressively lower levels of cyclin B1 expression (Figure 1F). Collectively, these results demonstrated the inhibitory effect of pristimerin on the proliferative capacity of TNBC cells. Considering the substantial inhibitory effects of pristimerin, we investigated its impact on TNBC cell apoptosis. Flow cytometry and Hochest 33342 stain were employed to assess the apoptotic rate in TNBC cells. As depicted in Figure 1E and Figure S1B (Supporting Information), pristimerin treatment significantly increased apoptosis in TNBC cells. Additionally, pristimerin treatment resulted in elevated expression of the pro‐apoptotic protein Bcl‐2 Associated X‐protein (Bax), cleaved poly ADP‐ribose polymerase (PARP) and cleaved caspase‐3, whereas decreased expression of the anti‐apoptotic protein B‐cell lymphoma 2 (Bcl‐2) (Figure 1G). Overall, our results highlight the significant anticancer efficacy of pristimerin in TNBC proliferation.

### 2.2 Autophagy Contributes to the Anti‐Tumor Activity of Pristimerin in TNBC

**Figure 2:**
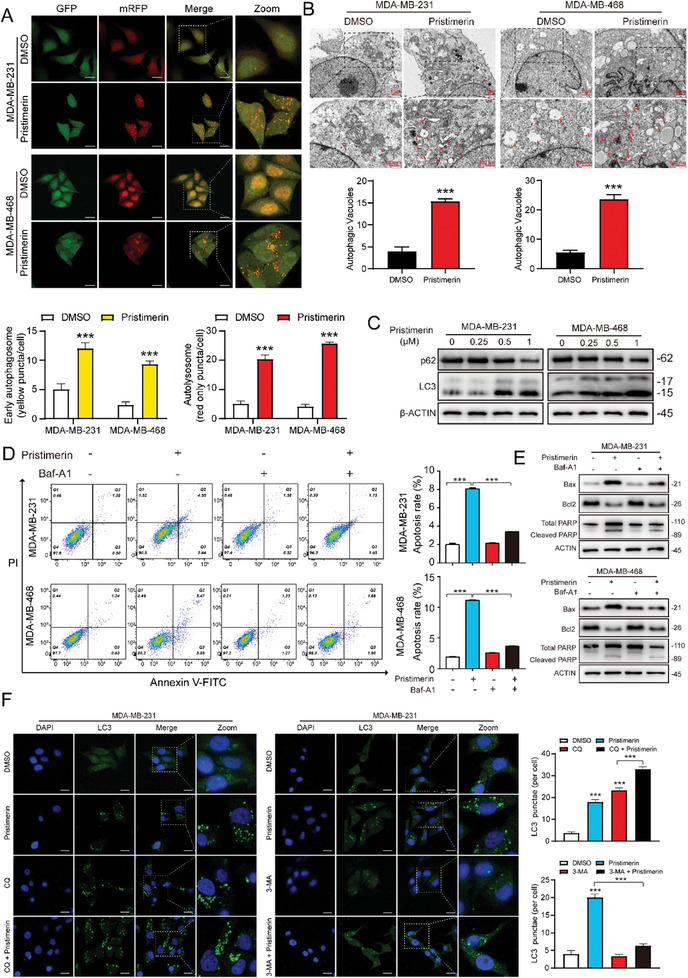
Autophagy contributes to the anti‐tumor activity of pristimerin in TNBC. (A) Representative images of early autophagosomes (yellow, GFP^+^ RFP^+^ puncta) and late autolysosomes (red, GFP^−^ RFP^+^ puncta) in TNBC cells treated with pristimerin for 48 h. Scale bar = 20 µm (B) Representative images of the autophagic structures (red arrows) of TNBC cells treated with pristimerin for 48 h. The number of autophagic vacuoles per area was quantified (*n* = 3). (C) The expression levels of p62 and LC3 were detected by Western blot after cells were treated with pristimerin for 48 h. (D,E) The apoptosis rate and expression of apoptotic markers in TNBC cells treated with pristimerin, with or without pretreated with 0.5 nm Baf‐A1 for 12 h, were detected by flow cytometry (D) and Western blot (E). (F) MDA‐MB‐231 cells were treated with pristimerin, with or without pretreated with 20 µm CQ or 1 mm 3‐MA for 12 h, and then the LC3 puncta were examined by immunofluorescence. Scale bar = 20 µm. Bars, SDs; ^*^0.01 < *p* < 0.05, ^**^0.001 < *p* < 0.01, and ^***^ *p* < 0.001.

To comprehensively verify whether other cell death forms are involved in pristimerin‐induced cell death, we used a panel of cell death inhibitors to rescue the cell viability under pristimerin treatment including copper chelator (tetrathiomolybdate, TTM), iron chelator (ferrostatin‐1, Fer‐1), autophagy inhibitor (bafilomycin A1, Baf‐A1), and apoptosis inhibitor (Z‐VAD‐FMK, ZMK). Our results indicated that the autophagy inhibitor and apoptosis inhibitor partially restored pristimerin‐induced cell death, while the copper and iron chelators had no significant effect (Figure S2A, Supporting Information). In fact, a body of evidence has demonstrated that the correlation between apoptosis and autophagy is intricate, and they can exhibit cooperative or antagonistic interactions. Here, we would like to investigate whether pristimerin‐induced apoptosis in TNBC cells is associated with autophagy. Therefore, GFP‐RFP‐LC3 lentivirus was used to infect MDA‐MB‐231 and MDA‐MB‐468 cells to observe the distribution of early autophagosomes (yellow puncta, GFP^+^ and RFP^+^) and late autolysosomes (red puncta, GFP^−^ and RFP^+^). The results obtained from confocal indicated a notable increase in both early autophagosomes and late autolysosomes following pristimerin treatment (**Figure** **2**A). More importantly, transmission electron microscope (TEM) images showed more autophagic vacuoles after treatment with pristimerin for 24 h (Figure 2B). Furthermore, Western blot analysis revealed that pristimerin facilitated the conversion of LC3‐I to LC3‐II while reducing the expression of sequestosome 1 (p62) in a concentration‐dependent manner (Figure 2C). In summary, these findings strongly suggested that pristimerin stimulated the accumulation of autophagosomes in TNBC cells.

To further validate the effect of autophagy in the induction of apoptosis, rescue experiments were conducted by employing the autophagy inhibitor Baf‐A1. As shown in Figure S2B,C (Supporting Information), the suppression of cell proliferation and colony formation caused by pristimerin were partially restored by Baf‐A1. Additionally, Baf‐A1 treatment significantly attenuated TNBC apoptosis induced by pristimerin (Figure 2D,E). Taken together, pristimerin exerted its anti‐tumor activity by activating cellular autophagy.

Next, given that autophagy is a dynamic process with multiple steps, we employed two autophagy inhibitors to further investigate this process. The first inhibitor is chloroquine (CQ), which serves as a late‐stage autophagy inhibitor. It functions by preventing the fusion of autophagosomes with lysosomes, thereby blocking the degradation of autophagosomes. Therefore, if autophagy initiation is activated, the blockade of autophagy by CQ would result in a further accumulation of LC3 puncta. The second inhibitor is 3‐methyladenine (3‐MA), an early‐stage autophagy inhibitor that can suppress the initial activation of autophagy. As shown in Figure 2F and Figure S2D,E (Supporting Information), we observed that upon blocking the degradation of autophagosomes with CQ, treatment with pristimerin led to an increase in LC3 puncta. However, when early‐stage autophagy was inhibited with 3‐MA, pristimerin did not promote the formation of LC3 puncta. These findings indicate that the accumulation of autophagosomes induced by pristimerin in TNBC cells is due to the activation of the initiation phase of autophagy, rather than a blockade of the degradation stage of autophagy.

### 2.3 Pristimerin Induces Autophagy Through the Activation of the MAPK/ERK Pathway

**Figure 3:**
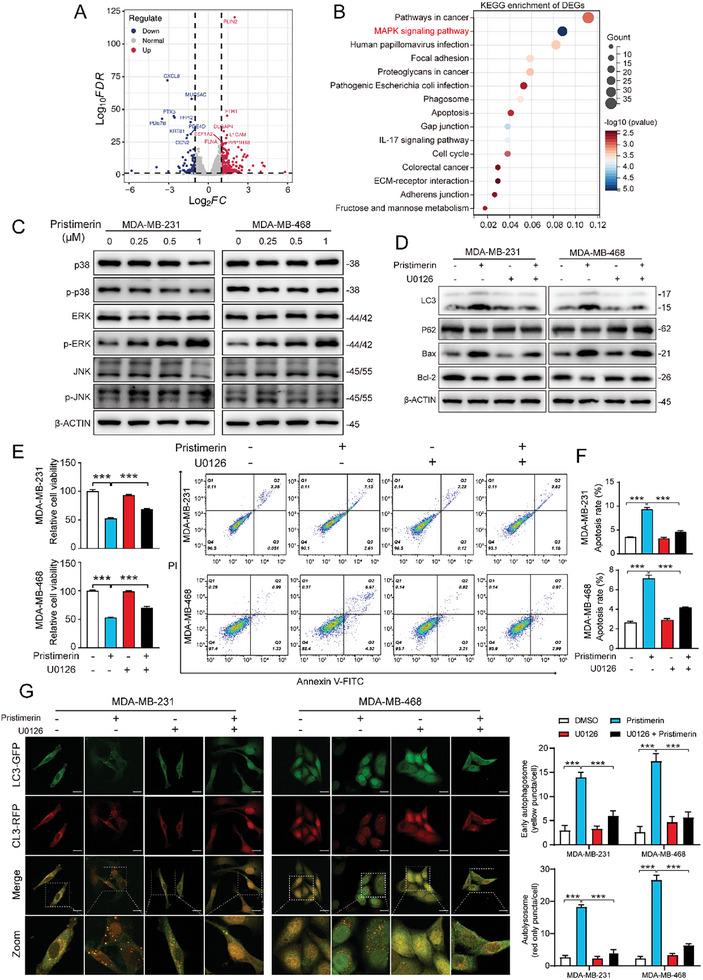
Pristimerin induces autophagy through the activation of the ERK pathway. (A) Volcano plot of DEGs (with fold change ≥ 1.5). (B) The top 15 pathways resulting from KEGG enrichment of DEGs. (C) Western blot showing the expression of MAPK pathway‐related proteins including p38, p‐p38, ERK, p‐ERK, JNK, and p‐JNK in TNBC cells after pristimerin treatment for 48 h. (D) The expression levels of autophagy‐related proteins p62 and LC3 as well as apoptosis‐related proteins Bax and Bcl‐2 were detected by Western blot after TNBC cells were treated with pristimerin for 48 h with or without the pretreated of ERK inhibitor U0126 (10 µm) for 2 h. (E,F) Cell viability and apoptosis rate were detected by CCK‐8 (E) and flow cytometry (F) after TNBC cells were treated with pristimerin for 48 h with or without the pretreated of U0126 (10 µm) for 2 h. (G) Representative images of autophagosomes and autolysosomes in TNBC cells treated with pristimerin for 48 h with or without the pretreated of U0126 (10 µm) for 2 h. Scale bar = 20 µm Bars, SDs; ^*^0.01 < *p* < 0.05, ^**^0.001 < *p* < 0.01, and ^***^ *p* < 0.001.

To elucidate the underlying mechanism of pristimerin‐induced cell autophagy, we compared the transcript expression profiles of MDA‐MB‐231 cells with pristimerin or DMSO treatment for 48 h by RNA sequencing (RNA‐seq). With the differential fold threshold set to 1.5, a total of 973 differentially expressed genes (DEGs) were generated, of which 636 up‐regulated and 337 down‐regulated (**Figure** **3**A; table S1, Supporting Information). Then, we conducted gene ontology (GO) enrichment analysis to elucidate the biological functions influenced by pristimerin. The results indicated that several biological processes related to cell death, including autophagy, cell cycle arrest, DNA damage, and apoptosis, all exhibited statistically significant differences. These findings are highly consistent with our previous conclusions (Figure S3A, Supporting Information). To further investigate the potential pathways involved, we conducted Kyoto encyclopedia of genes and genomes (KEGG) enrichment analysis and found the involvement of the MAPK pathway, a classical pathway known to be associated with cellular autophagy (Figure 3B). Therefore, we detected the activation of three classical pathways within the MAPK pathway. Notably, pristimerin treatment significantly activated the ERK MAPK pathway, as evidenced by a notable upregulation of p‐ERK expression, while the c‐Jun N‐terminal kinases (JNK) and p‐38 MAPK pathways were unaffected (Figure 3C).

To verify that the autophagic cell death induced by pristimerin was indeed modulated by the activation of the ERK MAPK pathway, we applied the ERK pathway inhibitor U0126. As anticipated, the inhibition of the ERK pathway by U0126 partially counteracted the anticancer activity of pristimerin, as evidenced by the restored cell proliferation capacity and reduced apoptosis rate (Figure 3D–F). Furthermore, the LC3 puncta aggregation which was significantly increased after pristimerin treatment was also partially reduced with U0126 added (Figure 3G). These findings provided compelling evidence that the activation of the ERK pathway plays a critical role in the mechanism of pristimerin‐induced autophagic cell death.

### 2.4 Pristimerin Directly Targets HSPA8

**Figure 4:**
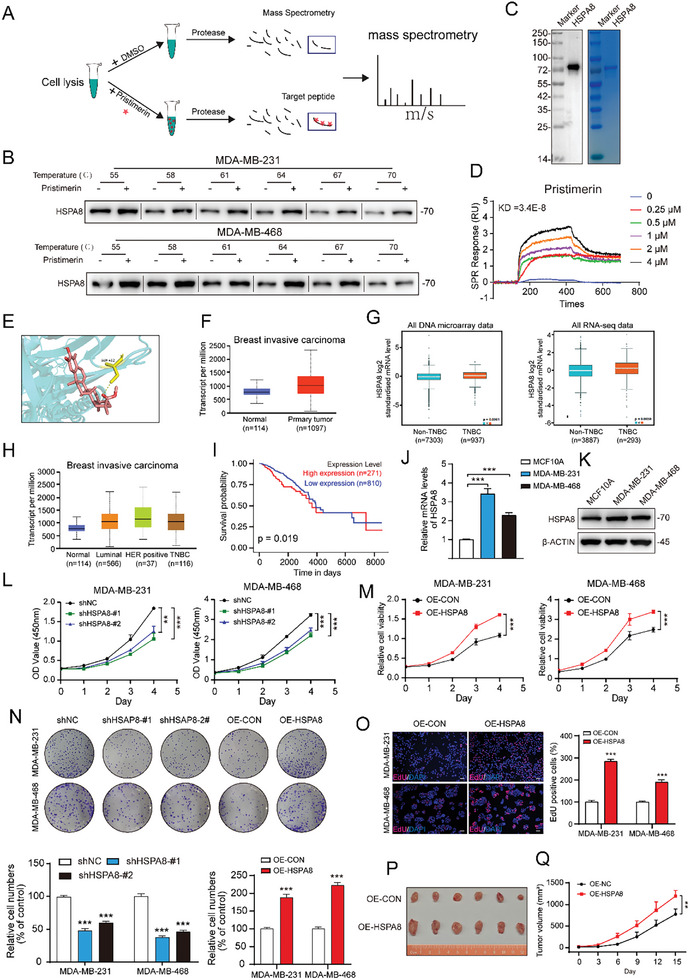
Pristimerin directly targets HSPA8. (A) Schematic depicting the rough flow of the DARTS‐MS assay. (B) Western blot of CETSA for HSPA8 with pristimerin or DMSO treatment for 1 h in TNBC cells. (C) SDS‐PEAG of the HSPA8 recombinant protein. (D) The binding affinity of HSPA8 recombinant protein with pristimerin was detected by SPR analysis. (E) The binding of HSPA8 with pristimerin was simulated by molecular docking. (F–H) The expression of HSPA8 in normal and tumor tissues were analyzed through UALCAN website (https://ualcan.path.uab.edu/index.html) and BCa Gene‐Expression Miner website (http://bcgenex.ico.unicancer.fr/BC‐GEM/GEM‐Accueil.php?js = 1). (I) The prognostic significance of HSPA8 in BCa (TCGA data) was assessed by Kaplan‐Meier analysis. (J,K) The expression of HSPA8 in normal breast epithelial cell lines MCF10A and TNBC cells lines were detected by qPCR and Western blot. (L,M) Cell viability of HSPA8 knockdown or overexpression cells was examined by CCK‐8. (N) The colony formation was evaluated in HSPA8 knockdown or overexpression cells. (O) The cell proliferation was detected by EdU assay in HSPA8 overexpression cells. Red: EdU‐positive cells. Scale bar = 100 µm. (P,Q) HSPA8 overexpression cell (4T1‐OE‐HSPA8) and vector cells (4T1‐OE‐NC) were injected subcutaneously into the left mammary fat pad of BALB/c mice, and then the tumor volume was recorded every three days (*n* = 6). Bars, SDs; ^*^0.01 < *p* < 0.05, ^**^0.001 < *p* < 0.01, and ^***^ *p* < 0.001.

Drug affinity response target stability (DARTS) is a novel technique that has been developed based on the principle that the binding of small molecule drugs to target proteins can reduce the sensitivity of these proteins to protease degradation. This technique is widely used in drug screening and target identification. In this study, the DARTS‐MS technique was employed to uncover the direct targets of pristimerin, as depicted in **Figure** **4**A. The mass spectrometry results revealed 67 proteins that could potentially serve as targets for pristimerin (Table S2, Supporting Information). To further investigate the binding potential of pristimerin to these proteins, we performed molecular docking and ranked them by docking score (Table S2, Supporting Information). In order to identify targets that are associated with autophagy, an extensive review of the literature was carried out to explore the relationship between the target proteins and autophagy (Table S2, Supporting Information). Among the top 20 proteins ranked by docking score, only TRPM2 and HSPA8 exhibited a close correlation with cellular autophagy, thus warranting their selection for experimental validation.^[^ ^20^, ^21^ ^]^ The cellular thermal shift assay (CETSA), which detects the binding of drugs to target proteins was employed. The results showed that pristimerin only enhanced the resistance of HSPA8 to heat denaturation, while it had no effect on the TRPM2 protein (Figure 4B; Figure S4A, Supporting Information). Significantly, surface plasmon resonance (SPR) experiments demonstrated a significant binding affinity between pristimerin and HSPA8 protein, with a dissociation constant (KD) of 3.4 × 10^−8^ (doxorubicin and docetaxel as negative control) (Figure 4C,D; Figure S4B, Supporting Information). Furthermore, the findings from molecular docking suggested that there was a possibility of pristimerin forming a hydrogen bond with the serine‐432 (SER‐432) residue of HSPA8 (Figure 4E). Therefore, we constructed the HSPA8^S432A^‐Flag mutant plasmid [Substitution of SER‐432 (S) for alanine‐432 (A)] and tested the binding between the exogenous mutant protein HSPA8^S432A^‐Flag and pristimerin by CETSA assay. As shown in Figure S4C (Supporting Information), pristimerin did not increase the resistance of the HSPA8^S432A^‐Flag protein to heat denaturation, which suggested that the SER‐432 site is crucial for the binding between HSPA8 and pristimerin (Figure S4C, Supporting Information). These observations strongly suggest a direct interaction between pristimerin and HSPA8 protein.

HSAP8 has been identified as a regulator of client proteins involved in multiple oncogenic processes, such as colon cancer, glioma, and endometrial cancer. While HSPA8 has been suggested as a potential prognostic indicator in TNBC,^[^ ^22^ ^]^ its specific role in TNBC is still not fully understood. We first explored the expression of HSPA8 in BCa. The expression of HSPA8 from the TCGA database analyzed on the UALCAN website showed that HSPA8 was upregulated in BCa and TNBC (Figure 4F,H). In addition, bc‐GenExMiner v5.0 is a published statistical mining tool specifically designed for the analysis of annotated BCa transcriptome data (including DNA microarrays [*n* = 11522] and RNA‐seq [*n* = 5023]). The results showed that HSPA8 was highly expressed in TNBC both in all DNA microarray data and DNA‐seq data (Figure 4G). More importantly, Kaplan–Meier survival analysis validated that the patients with high HSPA8 expression had shorter survival than the patients with low HSPA8 expression (Figure 4I). The qPCR and Western blot results also identified a higher expression of HSPA8 in TNBC cells compared to the mammary epithelial cell line MCF10A (Figure 4J,K).

Furthermore, we established HSPA8‐knockdown stable cell lines (named shNC, shHSPA8‐#1, and shHSPA8‐#2) and HSPA8‐overexpressing stable cell lines (named OE‐CON and OE‐HSPA8) for gain‐ and loss‐of‐function assays (Figure S4D–G, Supporting Information). The results from CCK‐8 and colony formation assays showed that the knockdown of HSPA8 significantly suppressed the proliferation and colony formation of TNBC cells, while HSPA8 overexpression markedly enhanced these behaviors (Figure 4L–N). In addition, EdU staining showed that HSPA8 overexpression increased the amount of newly synthesized DNA labeled with EdU, indicating significantly enhanced cell proliferation capacity of TNBC cells (Figure 4O). Moreover, we constructed an in‐situ animal model of breast cancer, and the results confirmed that HSPA8 overexpression promoted the growth of TNBC cells in vivo (Figure 4P,Q). Altogether, our findings revealed that HSPA8 contributed to the growth of TNBC cells, and pristimerin directly targeted HSPA8 protein.

### 2.5 The Anticancer Activity of Pristimerin Relies on HSPA8

**Figure 5:**
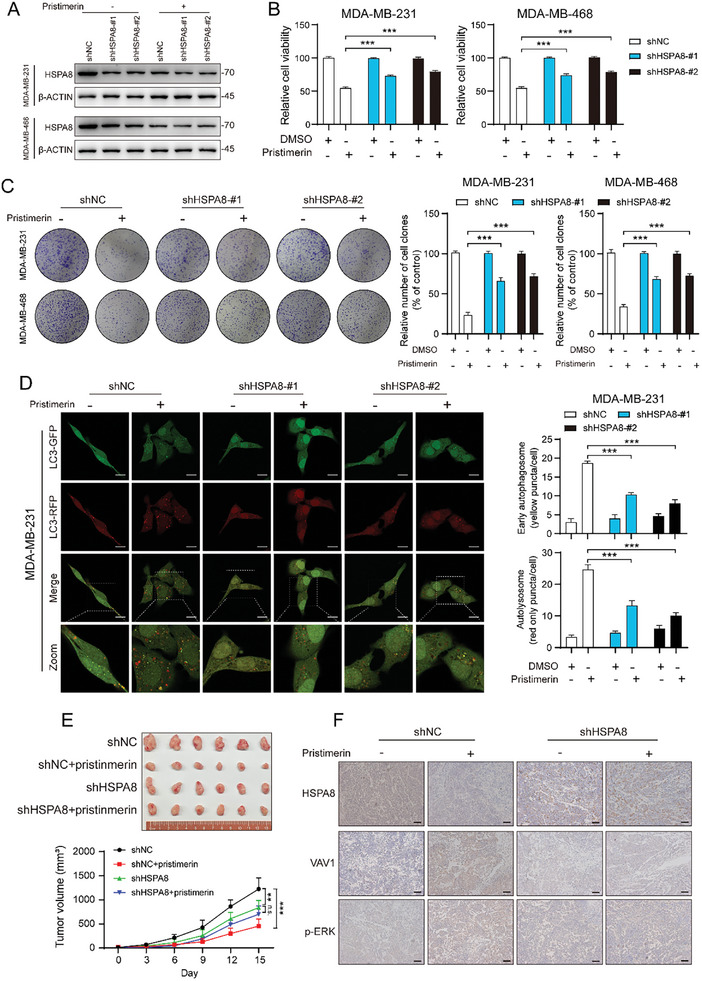
The anticancer activity of pristimerin relies on HSPA8. (A) The level of HSPA8 was determined in HSPA8 knockdown cells combined with pristimerin treatment. (B‐C) HSPA8 knockdown cells were treated with DMSO or pristimerin for 48 h, and then the cell viability and colony formation ability were assessed through CCK‐8 (B) and colony formation assays (C), respectively. (D) HSPA8 knockdown cells were treated with DMSO or pristimerin for 48 h, and then the autophagosomes and autolysosomes were examined by immunofluorescence. (E) HSPA8 knockout cells (4T1‐shNC and 4T1‐shHSPA8) were injected subcutaneously into the left breast fat pad of BALB/c mice and palpable tumors were allowed to develop for 7 days. Mice were randomly divided into four groups (*n* = 6) and treated with vehicle control (0.5% CMC‐Na) or 1 mg kg^−1^ pristimerin every other day. The tumor size was measured at indicated time intervals and tumors were imaged at the end of treatment. (F) The expressions of HSAP8, VAV1 and p‐ERK were detected by immunohistochemical assay. Scale bar = 50 µm Bars, SDs; ^*^0.01 < *p* < 0.05, ^**^0.001 < *p* < 0.01, and ^***^ *p* < 0.001.

We hypothesized that HSPA8 is a direct target of pristimerin‐induced autophagy and apoptosis in TNBC. To validate this hypothesis, we investigated the influence of pristimerin treatment on TNBC cell autophagy and proliferation under conditions of HSPA8 knockdown. First, we examined the changes in HSPA8 expression. The Western blot results showed that with the HSPA8 knockdown, pristimerin did not further significantly reduce the expression of HSPA8 (**Figure** **5**A). Then, the CCK‐8 and colony formation assays showed that HSPA8 knockdown partially mitigated the suppressive action of pristimerin on cell growth (Figure 5B,C). Additionally, elevated levels of early autophagosomes and late autolysosomes induced by pristimerin were significantly reduced after HSPA8 knockdown (Figure 5C; Figure S5A, Supporting Information). More importantly, the in vivo experiments showed that TNBC tumor xenografts were less sensitive to pristimerin when HSPA8 expression was downregulated (Figure 5E,F). Take together, all these findings pointed to a crucial role of HSPA8 in pristimerin‐induced TNBC inhibition.

### 2.6 Pristimerin Attenuates HSPA8‐Mediated VAV1 Degradation

**Figure 6:**
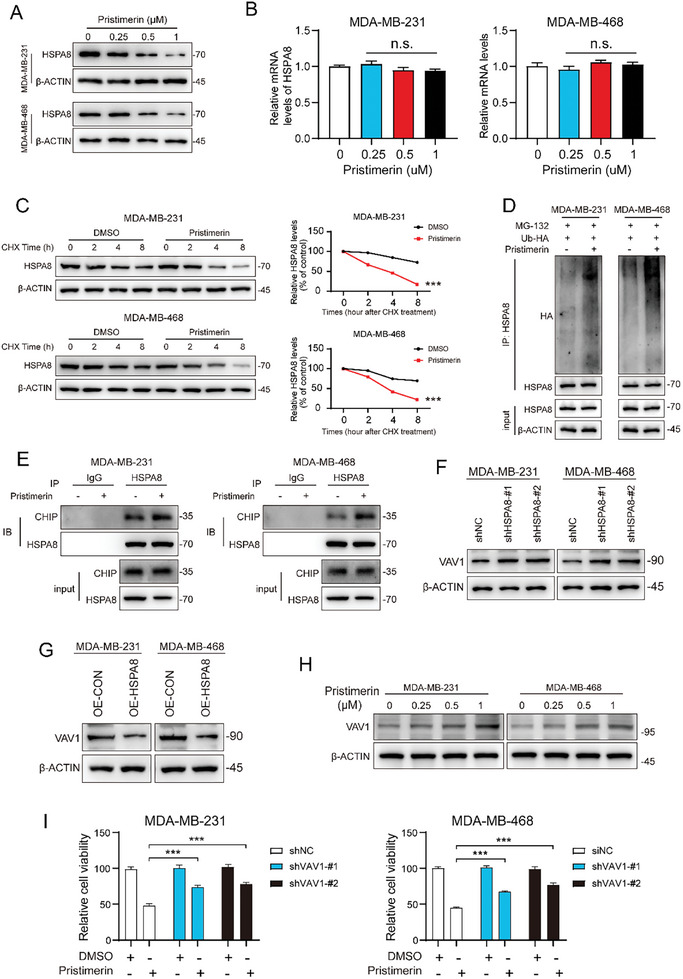
Pristimerin attenuates HSPA8‐mediated VAV1 degradation. (A,B) After treatment with different concentrations of pristimerin for 48 h, the HSPA8 expression was tested by Western blot (A) and qPCR (B). (C) Western blot was used to analyze HSPA8 expression in TNBC cells exposed to 100 µg mL^−1^ CHX in the presence or absence of pristimerin at the indicated times. (D) TNBC cells were transfected with Ub‐HA plasmid for 36 h, and then treated with 1 µm pristimerin and 5 µm MG‐132 for 24 h, followed by immunoprecipitation with anti‐HSPA8 antibody, and finally, HA levels were detected by Western blot. (E) HSPA8 overexpression cells were treated with pristimerin or DMSO for 48 h, cell lysis was collected and then immunoprecipitation with HSPA8 antibody to detect CHIP expression. (F,G) The expression of VAV1 in HSPA8 knockdown (F) or overexpression cells (G) were detected by Western blot. (H) The expression of VAV1 was determined after treating with pristimerin for 48 h. (I) The cell proliferation was detected in VAV1 knockdown cells combined with pristimerin treatment for 48 h. Bars, SDs; ^*^0.01 < *p* < 0.05, ^**^0.001 < *p* < 0.01, and ^***^ *p* < 0.001.

To further investigate the molecular mechanism by which pristimerin activates the ERK pathway to facilitate autophagy in TNBC cells, we detected the influence of pristimerin on HSPA8 expression. The findings demonstrated that the protein expression of HSPA8 was decreased in a dose‐dependent manner by pristimerin, whereas its transcription level remained unaffected (**Figure** **6**A,B). Therefore, we speculated that pristimerin promoted HSPA8 degradation. Then, we treated TNBC cells with cycloheheximide (CHX) to inhibit protein synthesis and observed the degradation of HSPA8 protein at different time points (0, 2, 4, and 8 h). The results demonstrated that pristimerin treatment significantly accelerated the degradation of HSPA8 compared to the DMSO group (Figure 6C). Previous studies have shown that HSPA8 is degraded through the ubiquitin‐proteasome pathway,^[^ ^23^ ^]^ and carboxyl terminus of Hsc70‐interactin protein (CHIP) E3 ubiquitin ligase is involved in mediating this process, which encourages us to study the degree of ubiquitination of HSPA8.^[^ ^24^, ^25^ ^]^ MG132, a proteasome inhibitor used to block the protein hydrolysis activity of the 26S proteasome complex, was therefore used in the next experiment. Unsurprisingly, our results showed that pristimerin promoted the binding of ubiquitin molecules to HSPA8, which significantly enhanced the ubiquitinated degradation of HSPA8 (Figure 6D). Additionally, pristimerin treatment notably augments the recruitment of the CHIP E3 ubiquitin ligase by HSPA8 (Figure 6E). Collectively, these results supported that pristimerin promotes CHIP‐mediated proteasomal degradation of HSPA8.

Given the crucial role of HSPA8 in CMA,^[^ ^26^ ^]^ we hypothesize that pristimerin promotes ERK pathway activation by inhibiting HSPA8 expression, thereby impeding the degradation of client proteins. To identify potential client proteins of HSPA8 involved in this process, we performed a literature review. We found that VAV1 is a client protein of HSPA8 which is degraded through the CMA pathway.^[^ ^27^ ^]^ VAV1 binds to HSPA8 via the 762–767 motif (QFPFKE) of VAV1, a process that maintains the degradation level of the VAV1 protein. That is, HSPA8 regulates the degradation of VAV1, and their expression is negatively correlated.^[^ ^27^ ^]^ Importantly, VAV1 is known to be an important protein that promotes ERK pathway activation.^[^ ^28^, ^29^ ^]^ Therefore, we first validated the expression between HSPA8 and VAV1. The results showed that HSPA8 knockdown increased the expression of VAV1, while HSPA8 overexpression decreased it (Figure 6F,G). This suggests a negative correlation between HSPA8 and VAV1, which is consistent with the conclusion that HSPA8 mediates the degradation of VAV1. Consequently, we next assessed the impact of pristimerin on VAV1 protein expression. As anticipated, pristimerin inhibited the degradation of VAV1 protein in a concentration‐dependent manner (Figure 6H). To further substantiate the critical role of VAV1 protein in pristimerin‐induced anticancer activity, we knocked down VAV1 expression to evaluate cell viability. It was shown that the pristimerin‐induced inhibition of cell proliferation and colony formation were partially attenuated by VAV1 knockdown (Figure 6I; Figure S6A,B, Supporting Information). Overall, our findings revealed that pristimerin attenuated HSPA8‐mediated degradation of VAV1, which in turn promoted ERK pathway activation.

### 2.7 Pristimerin Inhibits the Migration and Invasion of TNBC Cells

**Figure 7:**
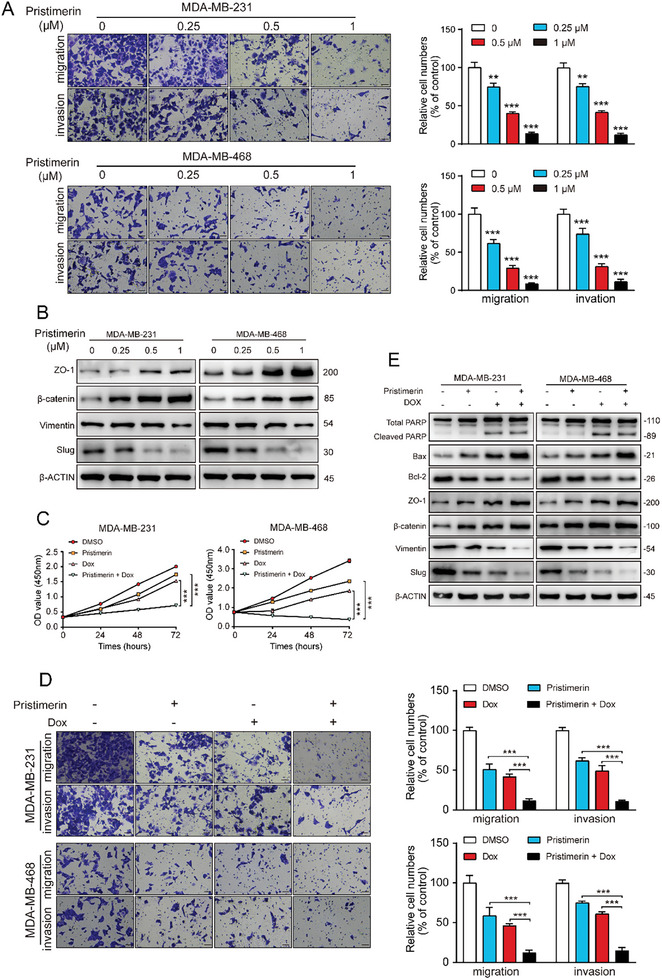
Pristimerin inhibits the migration and invasion of TNBC cells. (A) Cell migration and invasion ability were examined by Boyden chamber assay after treatment with pristimerin and 10 µg mL^−1^ Mitomycin C for 48 h. Scale bar = 100 µm (B) Cells were treated with DMSO or pristimerin for 48 h, and the levels of ZO‐1, β‐catenin, vimentin and slug were determined by Western blot. (C) Cells were treated with doxorubicin (0.3 µm) or pristimerin (0.25 µm) alone, or the combination of doxorubicin and pristimerin for 48 h, and the cell viability were determined by CCK‐8 assay. (D) In the presence of Mitomycin C, the cells were treated with doxorubicin (0.3 µm) or pristimerin (0.25 µm) alone, or the combination of doxorubicin and pristimerin for 48 h, and then the cell migration and invasion ability were examined. (E) Cells were treated with doxorubicin (0.3 µm) or pristimerin (0.25 µm) alone, or the combination of doxorubicin and pristimerin, and the levels of PARP, Bax, Bcl‐2, ZO‐1, β‐catenin, vimentin and slug were detected by Western blot. Bars, SDs; ^*^0.01 < *p* < 0.05, ^**^0.001 < *p* < 0.01, and ^***^ *p* < 0.001.

Next, we investigated the impact of pristimerin on the migration and invasion of TNBC cells. The Transwell assay demonstrated a significant inhibition of TNBC cell migration and invasion by pristimerin (**Figure** **7**A). This observation was further supported by increased protein expression of epithelial cell markers zonula occludens (ZO‐1) and β‐catenin, along with decreased protein expression of mesenchymal cell markers Vimentin and snail family transcriptional repressor 2 (Slug) (Figure 7B). Moreover, we explored the potential of pristimerin in overcoming chemoresistance. We assessed TNBC cell sensitivity to the first‐line chemotherapy drug doxorubicin in the presence or absence of pristimerin. Results from CCK‐8 and Transwell assays demonstrated that the combination of low‐dose doxorubicin and low‐dose pristimerin significantly inhibited TNBC cell proliferation, migration, and invasion compared to either treatment alone (Figure 7C,D). Consistently, this combination also led to the most significant changes in the apoptosis markers cleaved PARP, Bax, and Bcl‐2, as well as ZO‐1, β‐catenin, Vimentin, and Slug (Figure 7E). These findings collectively demonstrate that pristimerin can enhance the therapeutic sensitivity of TNBC cells to doxorubicin. Altogether, our results indicated that pristimerin has the potential to increase the therapeutic efficacy of doxorubicin in TNBC cells.

### 2.8 Pristimerin Suppresses the Growth of TNBC Cells In Vivo

**Figure 8:**
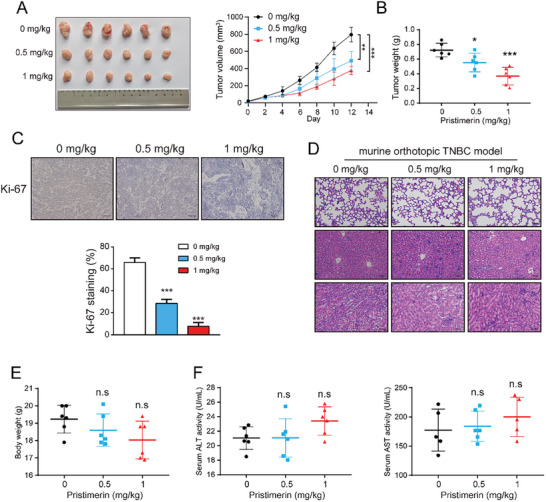
Pristimerin suppresses the growth of TNBC cells in vivo. (A,B) 4T1 cell was injected subcutaneously into the left breast fat pad of BALB/c mice and palpable tumors were allowed to develop for 7 days. Mice were randomly divided into three groups (*n* = 6) and treated with vehicle control (0.5% CMC‐Na), 0.5 mg kg^−1^ pristimerin or 1 mg kg^−1^ pristimerin every other day. The tumor size was measured at indicated time intervals and calculated; at the end of treatment, tumors were excised, imaged (A) and weighed (B). (C) Tumor tissues were fixed, sectioned, and placed on slides. Tumor specimens were subjected to IHC staining for Ki‐67. Scale bar = 50 µm (D) Hematoxylin and eosin (H&E) staining of lung, liver, and kidney specimens. Scale bar = 50 µm (E) The body weights of mice at the end of experiment. (F) The serum levels of ALT and AST in mice at the end of experiment (*n* = 6). Bars, SDs; ^*^0.01 < *p* < 0.05, ^**^0.001 < *p* < 0.01, and ^***^ *p* < 0.001.

To investigate the effect of pristimerin on TNBC growth in vivo, we established the murine orthotopic TNBC model (**Figure** **8**) and a subcutaneous xenograft tumor model (Figure S7, Supporting Information). The findings demonstrated that pristimerin effectively hindered the growth of TNBC, as evidenced by a reduction in tumor volume and weight (Figure 8A,B; Figure S7A,B, Supporting Information). Furthermore, immunohistochemistry and Western blot analysis were conducted to assess the expression levels of relevant proteins in the tumor tissue samples. Immunohistochemical results revealed that pristimerin inhibited Ki‐67 expression in a concentration‐dependent manner. (Figure 8C). Western blot revealed that pristimerin promoted the phosphorylation of ERK, as well as the occurrence of cellular autophagy and apoptosis (Figure S7C, Supporting Information). Importantly, no significant alterations in the pathological morphology of vital organs were observed (Figure 8D; Figure S7D, Supporting Information). Additionally, no notable differences were observed in animal body weight or the levels of serum alanine aminotransferase (ALT) and aspartate aminotransferase (AST) (Figure 8E,F; Figure S7E,F, Supporting Information). These findings suggested that pristimerin treatment had no significant toxic side effects on animals. In summary, our study provides evidence supporting the therapeutic efficacy and safety of pristimerin as an anticancer agent.

## 3 Discussion

TNBC, known for its unfavorable prognosis, has spurred the urgent need for the development of innovative targeted therapeutics. Our study introduces fresh evidence regarding the efficacy of pristimerin in treating TNBC. We conducted a comprehensive investigation to assess the effectiveness, safety, and potential mechanisms of pristimerin. Our findings indicate that pristimerin directly targets HSPA8 and promotes its ubiquitination and degradation. This subsequently inhibits the degradation of VAV1, a downstream client protein of HSPA8, leading to the activation of the ERK pathway and ultimately resulting in autophagy and apoptosis. Additionally, animal experimental results suggest that pristimerin exhibits no significant toxic effects on vital organs at doses lower than 1 mg kg^−1^.

Natural products have served as an important reservoir for the discovery and development of novel drugs due to their relatively low toxicity and their pharmacological attributes involving multiple components and targets. Prominent examples include paclitaxel and adriamycin, which have been successfully utilized in clinical therapy for numerous years. Research indicates that naturally occurring triterpenoids possess potential applications in anticancer, anti‐inflammatory, anti‐malarial, and insecticide treatments.^[^ ^30^, ^31^, ^32^ ^]^ Pristimerin, a quinonemethide triterpenoid derived from various plants belonging to *Celastraceae* and *Hippocrateaceae* families, such as *Maytenus heterophylla* ^[^ ^33^ ^]^ and *Celastrus aculeatus Merr*.,^[^ ^34^ ^]^ exhibits broad anticancer properties, as suggested by early investigations.^[^ ^14^, ^15^, ^16^ ^]^ However, the specific targets and molecular mechanisms of action for pristimerin remain unreported. Especially in terms of direct binding targets for pristimerin action, nothing has been reported so far.

Here, we demonstrated for the first time that pristimerin targeted the HSPA8 and promoted its ubiquitination and degradation. Interestingly, growing evidence suggests that HSPA8 is upregulated in various tumors and is associated with poor prognosis in patients.^[^ ^17^, ^18^, ^19^ ^]^ However, the role of HSPA8 in TNBC remains unclear. Our results indicated that silencing HSPA8 inhibited the proliferation and colony formation of TNBC cells. Furthermore, silencing HSPA8 attenuated the apoptotic effect induced by pristimerin, suggesting that the anticancer activity of pristimerin relies on HSPA8. More importantly, pristimerin promoted the ubiquitination and degradation of HSPA8 mediated by CHIP E3 ubiquitin ligase. It has been reported that HSPA8 mediates the degradation of unfolded or misfolded proteins by binding to exposed KFERQ amino acid motifs and delivering them to lysosomes.^[^ ^35^ ^]^ Among them, VAV1 is a client protein that has been validated to undergo degradation through the interaction with HSPA8 in the CMA pathway.^[^ ^27^ ^]^ Importantly, pristimerin increased the expression of VAV1 protein in a concentration‐dependent manner, and silencing VAV1 similarly weakened the anticancer activity of pristimerin. Additionally, although we discovered the SER‐432 residue of HSPA8 was crucial for the binding between HSPA8 and pristimerin, the crystal structure information is warranted.

Extensive research has been conducted on the role of autophagy in cancer, and it is widely recognized that autophagy plays a dual and contradictory role in tumor progression.^[^ ^36^ ^]^ On the one hand autophagy can provide the necessary nutrients and energy for tumor cells promoting tumor growth and progression.^[^ ^37^ ^]^ On the other hand, sustained autophagy stimulation can lead to depletion of organelles and key proteins, ultimately resulting in tumor cell death.^[^ ^38^, ^39^ ^]^ In our study, it was observed that pristimerin upregulated the expression of the autophagy marker protein LC3 while downregulating the expression of the autophagy substrate p62. Importantly, when TNBC cells were pretreated with the autophagy inhibitor Baf‐A1, the impact of pristimerin on cell proliferation and apoptosis was significantly diminished, suggesting that pristimerin induces TNBC cell apoptosis as an autophagy activator. Furthermore, the combination of the early autophagy inhibitor CQ and the late autophagy inhibitor 3‐MA revealed that the autophagosome accumulation induced by pristimerin in TNBC cells was due to activation of the initiation phase of autophagy rather than blocking the degradation phase of autophagy. This further clarifies the effect of pristimerin on autophagy flux.

This study revealed that pristimerin promoted autophagy by activating the MAPK/ERK pathway through RNA‐seq. The MAPK pathway includes three major branches: the ERK pathway, the JNK pathway, and the p38 pathway, which are closely interconnected and regulated in relation to cell autophagy.^[^ ^40^ ^]^ The ERK pathway can enhance the expression of autophagy genes, thereby activating the process of cell autophagy.^[^ ^41^, ^42^ ^]^ The JNK pathway and p38 MAPK pathway can regulate post‐translational modifications, thereby influencing autophagy progression.^[^ ^43^ ^]^ Conversely, cell autophagy can also regulate the activity of the MAPK pathway.^[^ ^44^ ^]^ Here, our results demonstrated that pristimerin selectively activated the ERK pathway, rather than the other two pathways. Importantly, the use of an ERK inhibitor U0126 prevented the anticancer activity of pristimerin, indicating the crucial role of ERK pathway activation in pristimerin‐mediated inhibition of TNBC progression. Additionally, cancer treatment failure is frequently attributed to tumor metastasis and drug resistance.^[^ ^45^, ^46^ ^]^ Here, we observed that pristimerin not only suppressed the migration and invasion of TNBC cells but also augmented the anti‐tumor efficacy of doxorubicin, suggesting its potential use in postoperative adjuvant therapy. However, further investigation is required to determine whether pristimerin can inhibit TNBC cell metastasis and increase the sensitivity of TNBC cells to doxorubicin in vivo.

**Figure 9:**
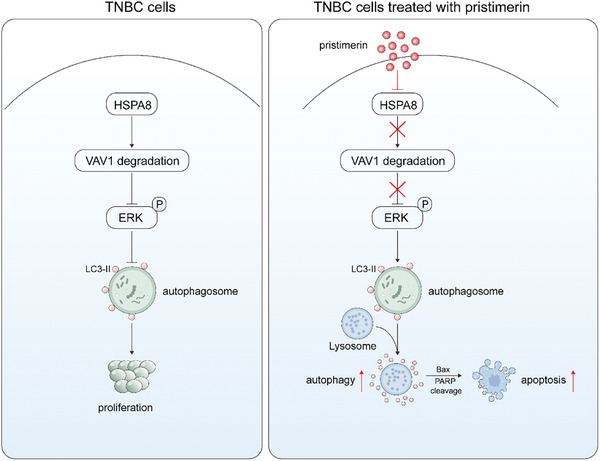
Schematic representation of the mechanisms by which pristimerin inhibits TNBC progression. In TNBC cells, HSPA8 facilitates the degradation of VAV1, thereby inhibiting the activation of the ERK pathway, suppressing cellular autophagy, and regulating the balance between cell proliferation and apoptosis. However, in the presence of pristimerin, it directly targets HSPA8, promoting its ubiquitination and degradation. This further inhibits the degradation of the downstream client protein VAV1, leading to the activation of the ERK pathway and mediating cellular autophagy and apoptosis. Ultimately, this leads to the suppression of cell proliferation, cell migration, and drug resistance.

To summarize, we uncovered that pristimerin has promising potential as a natural anticancer drug that can suppress the growth and metastasis of TNBC cells while increasing their responsiveness to doxorubicin treatment. The underlying mechanism involves that pristimerin directly targets and promotes the degradation of the HSPA8 protein, which activates the VAV1/ERK signaling pathway, resulting in cell autophagy and apoptosis (**Figure** **9**). These findings unveil the therapeutic targets and mechanisms of action of pristimerin, highlighting its potential application in the treatment of TNBC.

## 4 Experimental Section

### Cell Culture and Agents

Cells were supplemented with 10% fetal bovine serum (FBS, ExCelll bio, Shanghai) in a humidified atmosphere of 5% CO_2_ at 37 °C. Pristimerin, U0126, and MG‐132 were acquired from TargetMol (Boston, MA). Bafilomycin A1, Chloroquine, and Mitomycin C were purchased from MedChemExpress. Doxorubicin, docetaxel and cycloheximide (CHX) were obtained from Selleck Chemicals (Huston, TX, USA). Ferrostatin‐1, Z‐VAD‐FMK, tetrathiomolybdate were purchased from Ambeed Corporation (Huston, TX, USA).

### Plasmid and Transfection

The shRNAs for silencing HSPA8 and VAV1 were purchased from TsingKe Biological Technology (Shanghai, China). The sequences are as follows: shHSPA8‐#1 (5ʹ‐GCAACTGTTGAAGATGAGAAA‐3ʹ), shHSPA8‐#2 (5ʹ‐GCTGGTCTCAATGTACTTAGA‐3ʹ), shVAV1‐#1 (sense 5ʹ‐ CCTGCTACCCCATGCCATC ‐3ʹ), shVAV1‐#2 (sense 5ʹ‐ TCTCTACCAGGTCTTCATC ‐3ʹ). Transfection was conducted with the Lipofectamine 3000 kit (Thermo Fisher Scientific).

### Cell Viability Assay

Briefly, cells were seeded in a 96‐well plate and treated with or without specified drugs for a designated time. For detection, 10 µL CCK‐8 (APExBIO, Houston, USA) was added and incubated at 37 °C for 2 h. Results were obtained by measuring the absorbance at 450 nm with a microplate reader (Tecan infinite M200).

### Colony Formation Assay

Cells were plated in 6‐well plates at a density of 1000 cells per well. They were then exposed to the designated drugs for 10 days, during which the culture medium was refreshed every 2–3 days. After the completion of the experiment, the cells were fixed with 4% paraformaldehyde for 15 min and subsequently stained with 1% violet crystal.

### Migration and Invasion Assays

The cellular motility was examined using Boyden chambers (8 µm pore size, BD Biosciences, Bedford, MA, USA). In brief, cells suspended in medium without FBS were placed in the upper chamber for the migration assay or in the chamber coated with matrigel (BD Biosciences, Bedford, MA, USA) for the invasion assay. 20% FBS medium were added in lower chambers. To eliminate the potential effects of cell proliferation, cells were treated with 10 µg mL^−1^ Mitomycin C together with pristimerin. 48 h later, cells residing on the upper membrane were eliminated, while the cells that had migrated were fixed and stained. Subsequently, the migrated cells were visualized under the microscope.

### Cell Cycle and Apoptosis Assays

Cells were seeded in 6‐well plate and treated with or without specified drugs. 48 h later, cells were stained with annexin V and propidium iodide (PI) for 30 min (KeyGEN BioTHCE) for apoptosis analysis. For cell cycle analysis, cells were first fixed overnight in 70% cold ethanol and then stained with PI for 30 min (KeyGEN BioTHCE). Analyses were performed with the FACScan flow cytometer (BD Biosciences, San Jose, CA, USA).

### Western Blot

Total protein extracted from RIPA lysate was quantified by BCA quantification kit (Thermo Fisher Scientific), and an aliquot of protein sample (20–30 ug) was subjected to sodium dodecyl sulfate‐polyacrylamide gel electrophoresis (SDS‐PAGE) gels. Following electrophoresis, the proteins were transferred onto PVDF membranes (Millipore, Bedford, MA). The membrane was blocked with 5% skim milk for 1.5 h, followed by incubation with the primary antibody overnight at 4 °C. Afterward, the membrane was washed three times with TBST (Tris‐buffered saline with Tween‐20) and then incubated with the secondary antibody. Signals were measured with Clarity Western ECL substrate (Abbkine Scientific Co., Ltd, China). The expression of β‐ACTIN was used as an internal reference. The antibodies used in the experiment include: β‐ACTIN (#13E5, 1:1000, CST), Bax (A19684, 1:1000, ABclonal), Bcl‐2 (A19693, 1:1000, ABcloal), PARP1 (13371‐1‐AP, 1:1000, PTG), Cleaved Caspase 3 (25128‐1‐AP, 1:1000, PTG), Cyclin B1 (PTM‐6659, 1:1000, PTM Bio), p‐CDK1 (4539T, 1:1000, CST), CDK1 (sc‐54, 1:1000, Santa Cruz), p62 (18420‐1‐AP, 1:4000, PTG), LC3 (14600‐1‐AP, 1:1000, PTG), p38 (8690T, 1:1000, CST), p‐p38 (4511T, 1:1000, CST), ERK (4695T, 1:1000, CST), p‐ERK (4370T, 1:1000, CST), JNK (66210‐1‐Ig, 1:3000, PTG), p‐JNK (80024‐1‐RR, 1:1000, PTG), HSPA8 (10654‐1‐AP, 1:1000, PTG), TRPM2 (21842‐1‐AP, 1:500, PTG), VAV1 (4657T, 1:1000, CST), ZO‐1 (21773‐1‐AP, 1:5000, PTG), β‐catenin (51067‐2‐AP, 1:5000, PTG), Vimentin (A11952, 1:1000, ABcloal), Slug (sc‐166476, 1:1000, Santa Cruz). HA tag (51064‐2‐AP, 1:5000, PTG).

### qPCR

Total RNA Isolation Reagent Kit (Magen, China) was employed to obtain RNA. Subsequently, the RNA was converted to cDNA using the reverse transcription kit (TransGen Biotech, Beijing, China). Quantitative PCR (qPCR) was conducted by the SYBR Green PCR kit (Vazyme Biotech Co., Ltd). The primer sequences employed for the reactions are listed below: HSPA8 (Forward primer: 5ʹ – ACCTACTCTTGTGTGGGTGTT ‐ 3ʹ, Reverse primer: 5ʹ – GACATAGCTTGGAGTGGTTCG ‐ 3ʹ), VAV1 (Forward primer: 5ʹ – CAACCTGCGTGAGGTCAAC ‐ 3ʹ, Reverse primer: 5ʹ – ACCTTGCCAAAATCCTGCACA ‐ 3ʹ), GAPDH (Forward primer: 5ʹ – CCATCACCATCTTCCAGGAG ‐ 3ʹ, Reverse primer: 5ʹ – ATGATGACCCTTTTGGCTCC ‐ 3ʹ).

### Immunofluorescence Staining

Briefly, the cells were fixed using 4% paraformaldehyde, followed by the application of an immunostaining permeabilization agent (P0096, Beyotime, China) for 20 min and blocking with 5% goat serum for 30 min. LC3 antibody (14600‐1‐AP, 1:200, PTG) was then added and incubated overnight at 4 °C. Subsequently, the cells were incubated with CoraLite488‐conjugated Goat Anti‐Rabbit IgG secondary antibody (SA00013‐2, 1:200, PTG) and counterstained with DAPI (P0131, Beyotime, China). Images were captured under the same excitation light intensity.

### Immunohistochemical assay

The paraffin‐embedded slices were subjected to deparaffinization using xylene and alcohol, followed by antigen retrieval using 0.1 m citrate buffer (pH 6.0). Subsequently, the slices were treated with 3% hydrogen peroxide for 30 min and blocked with 5% goat serum for 1 h. For immunostaining, tissue slices were incubated with Ki‐67 (27309‐1‐AP, 1:200, PTG), Bax (A19684, 1:200, ABclonal), Bcl‐2 (A19693, 1:200, ABcloal), E‐cadherin (A20798, 1:200, ABclonal) and β‐catenin (51067‐2‐AP, 1:1000, PTG) antibodies. On the following day, a peroxidase‐conjugated avidin‐biotin complex and 3, 3‐diaminobenzidine (DAKO) were utilized as the chromogen, and the slices were then counterstained with hematoxylin.

### Coimmunoprecipitation Assay

The cell lysate was prepared using IP lysis buffer (P0013, Beyotime, China), followed by overnight incubation at 4 °C with anti‐HSPA8 antibody. Subsequently, protein A/G magnetic beads (HY‐K0202, MedChemExpress) were added for 4 h. After washing, Western blot analysis was performed.

### RNA‐seq and KEGG Enrichment Analysis

The cells were treated with pristimerin or DMSO for 48 h. Total RNA extraction was performed following the instructions provided by the Total RNA Isolation Reagent Kit (Magen, China), and the samples were then sent to Annoroad Gene Technology (Beijing, Co., Ltd) for RNA sequencing analysis. Here, genes with a fold change ≥ 1.5 and a *p* value < 0.05 were defined as differentially expressed genes. KEGG enrichment analysis was conducted using the Sangerbox online platform (http://sangerbox.com/login.html).

### Molecular Docking

The structures of protein were collected from the Protein Data Bank (https://www.rcsb.org/). Molecular docking was performed using AutoDock Tools^[^ ^47^ ^]^ and AutoDock Vina,^[^ ^48^ ^]^ with a docking seed set to 10 000, an energy range set to 3, and an exhaustiveness set to 4. The docking results were evaluated based on affinity (kcal/mol) index, and visualization was conducted using pyMOL.

### Drug Affinity Responsive Target Stability and Mass Spectrometry (DARTS‐MS)

The collected cell lysate was divided into two equal parts, with one part treated with pristimerin and the other part treated with DMSO. After incubation for 30 min at room temperature, protein was digested by adding proteinase K (ST533, Beyotime, China) for 5 min, and then terminated by adding phenylmethanesulfonyl fluoride (PMSF). Mass spectrometry analysis was performed at Shanghai Applied Protein Technology (Shanghai, China).

### Cellular Thermal Shift Assay (CETSA)

The harvested cells were resuspended in 1% PMSF PBS and underwent multiple freeze‐thaw cycles in liquid nitrogen to induce cell lysis. The lysate was then divided into two equal parts, with one part treated with pristimerin and the other part treated with an equal volume of DMSO, and incubated for 1 h at room temperature. Afterward, the supernatant was further divided into ten equal portions and subjected to heating at temperatures of 43, 46, 49, 52, 54, 57, 60, 63, 67, and 70 °C for 3 min each. Finally, the collected supernatant was analyzed by Western blot.

### Surface Plasmon Resonance (SPR)

The PlexArray HT A100 system was employed to measure the binding affinity between HSPA8 recombinant protein (MedChemExpress) and pristimerin/doxorubicin/ docetaxel. In summary, 1 µg of HSPA8 recombinant protein was fixed onto an activated 3‐D photocrosslinked chip. Subsequently, various concentrations of the drugs were passed over the chip surface at a specific flow rate in order to assess the binding affinity.

### Transmission Electron Microscopy (TEM)

The treated TNBC cells were incubated with a pre‐chilled 2% glutaraldehyde solution for 2 h at 4 °C to fix the cell pellet. Dehydration is carried out through a graded ethanol series: 50% for 15 min, 70% for 15 min, 80% for 15 min, 90% for 15 min, 95% for 15 min, followed by two changes of 100% ethanol for 15 min each, and finally two changes of acetone for 15 min. Then, the cells were embedded in spurr embedding kit, and ultrathin sections were prepared for observation under an electron microscope (HITACHI).

### Nude Mice Xenograft Model

6 weeks female nude mice and BALB/c mice were housed under standard conditions and cared for according to the guidelines for animal welfare. The animal experiment was ethically approved by the Ethics Committee of Southern Medical University. To establish subcutaneous xenograft tumor model, a suspension of 2 × 10^6^ MDA‐MB‐468 cells were subcutaneously injected into the flanks of nude mice. To establish murine orthotopic TNBC model, a suspension of 1 × 10^6^ 4T1 cells were subcutaneously injected into the left mammary fat pads of the BALB/c mice. After a week, the mice were randomly assigned to three groups (6 mice per group), including the high‐dose pristimerin group (1 mg kg^−1^), low‐dose pristimerin group (0.5 mg kg^−1^), and vehicle control group (0.5% CMC‐Na). Administration was performed every other day by intraperitoneal injection for a total of 7 times. Finally, tumor tissues as well as major organs (liver, kidney, and lung) were collected for further pathological analysis. Tumor volume was calculated as: *V* = (length × width^2^) / 2.

### Statistical Analysis

The mean ± SD values were used to present all the data, which were representative of the results obtained from at least three independent experiments. GraphPad Prism 8.0 was used for all statistical analyses. Statistical significance was indicated as ^*^for 0.01 < *p* < 0.05, ^**^for 0.001 < *p* < 0.01, and ^***^for *p* < 0.001.

### Ethics Approval and Consent to Participate

The experimental protocol for animal studies was reviewed and approved by the institutional animal care and use committee of Southern Medical University (SMUL202403019).

## Conflict of Interest

The authors declare no conflict of interest.

## Author Contributions

Q.‐W.L. acquired funding acquisition, analysis, interpretation of the data, statistical analysis, and wrote the final manuscript. Q.‐L.F., J.‐Y.C. acquired funding acquisition of the data, analysis, interpretation of the data. J.‐X.L., Q.L., Y.‐P.C., H.‐T.W., A.‐Q.X., S.W. performed technical, materials support. Y.L. performed molecular docking. D.‐G.G., A.‐P.L. acquired funding acquisition, study concept, design, study supervision. All authors edited, approved the final manuscript.

## Supporting information



Supporting Information

Supplemental Table 1

Supplemental Table 2

## Data Availability

The data that support the findings of this study are available from the corresponding author upon reasonable request.
