# Supplementary material for: Pristimerin Promotes Ubiquitination of HSPA8 and Activates the VAV1/ERK Pathway to Suppress TNBC Proliferation
Source: Adv Sci (Weinh). 2025 Jan 15;12(10):2413174. doi: 10.1002/advs.202413174 (PMC11904939; doi:10.1002/advs.202413174)
Supplement: Supplementary file 1 — Supporting Information [file ADVS-12-2413174-s003.docx]

**Supplementary Materials for**

Pristimerin promotes ubiquitination of HSPA8 and activates the VAV1/ERK pathway to suppress TNBC proliferation

Qin-Wen Liu ^1,2,3#^, Qi-Ling Fan ^2#^, Jia-Ying Chen ^4#^, Jing-Xin Liu ^2^, Yi Li ^2^, Qian Luo ^2^, Yu-Peng Chen ^3,5^, Hang-Tian Wu ^6^, An-Qi Xu ^1^, Sheng Wang ^7^, Ai-Ping Lu ^3,5,8*^, Dao-Gang Guan ^2*^

**
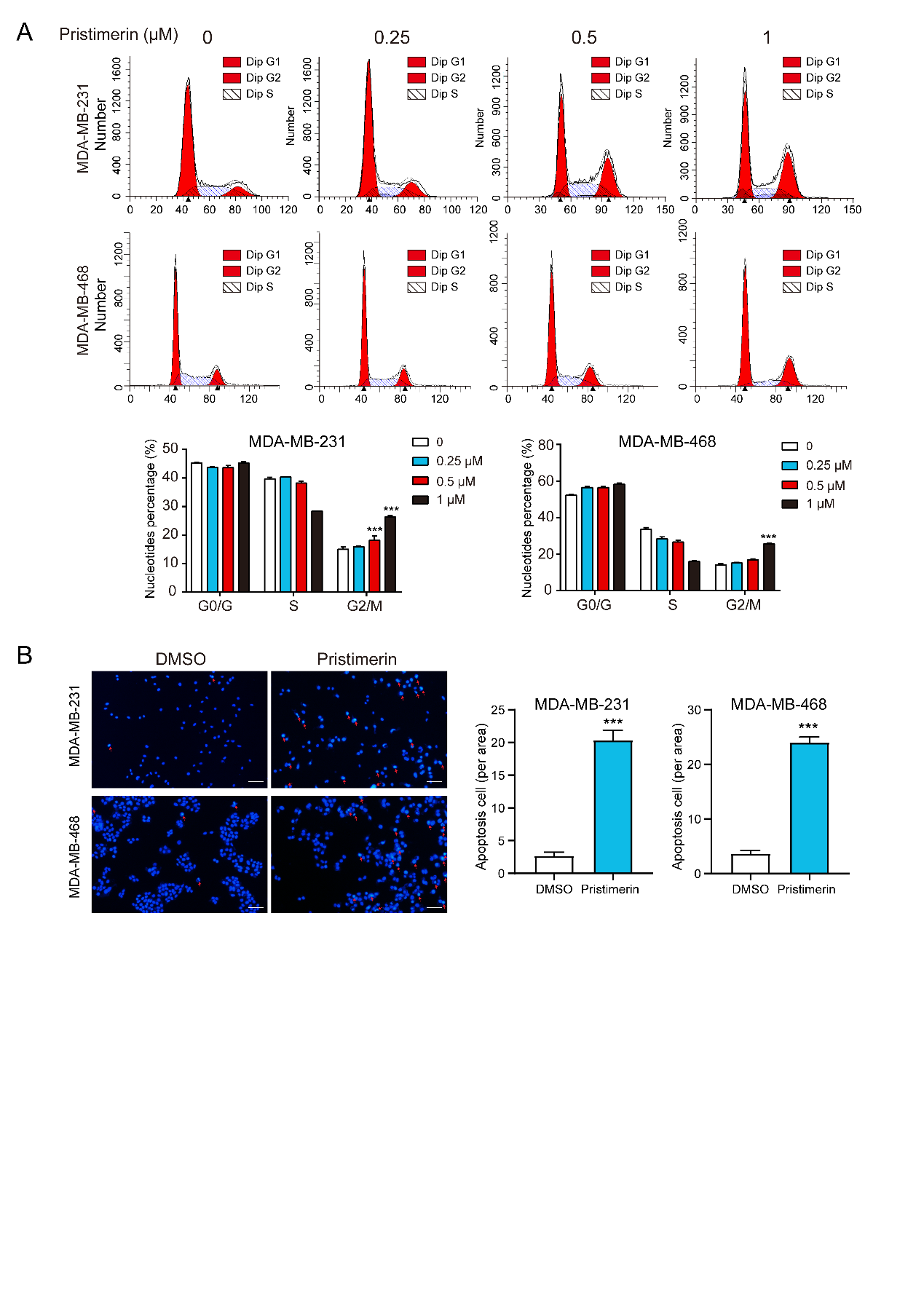
**

**Supplementary Figure S1.** (A) Cell cycle was analyzed using flow cytometry after the treatment of pristimerin for 48 h. (B) Cell apoptosis was analyzed via Hochest 33342 staining after the treatment of pristimerin for 48 h. Apoptotic nuclei are marked with red arrows. Scale bar = 100 μm. Bars, SDs; * 0.01 < *P* < 0.05, ** 0.001 < *P* < 0.01, and *** *P* < 0.001.


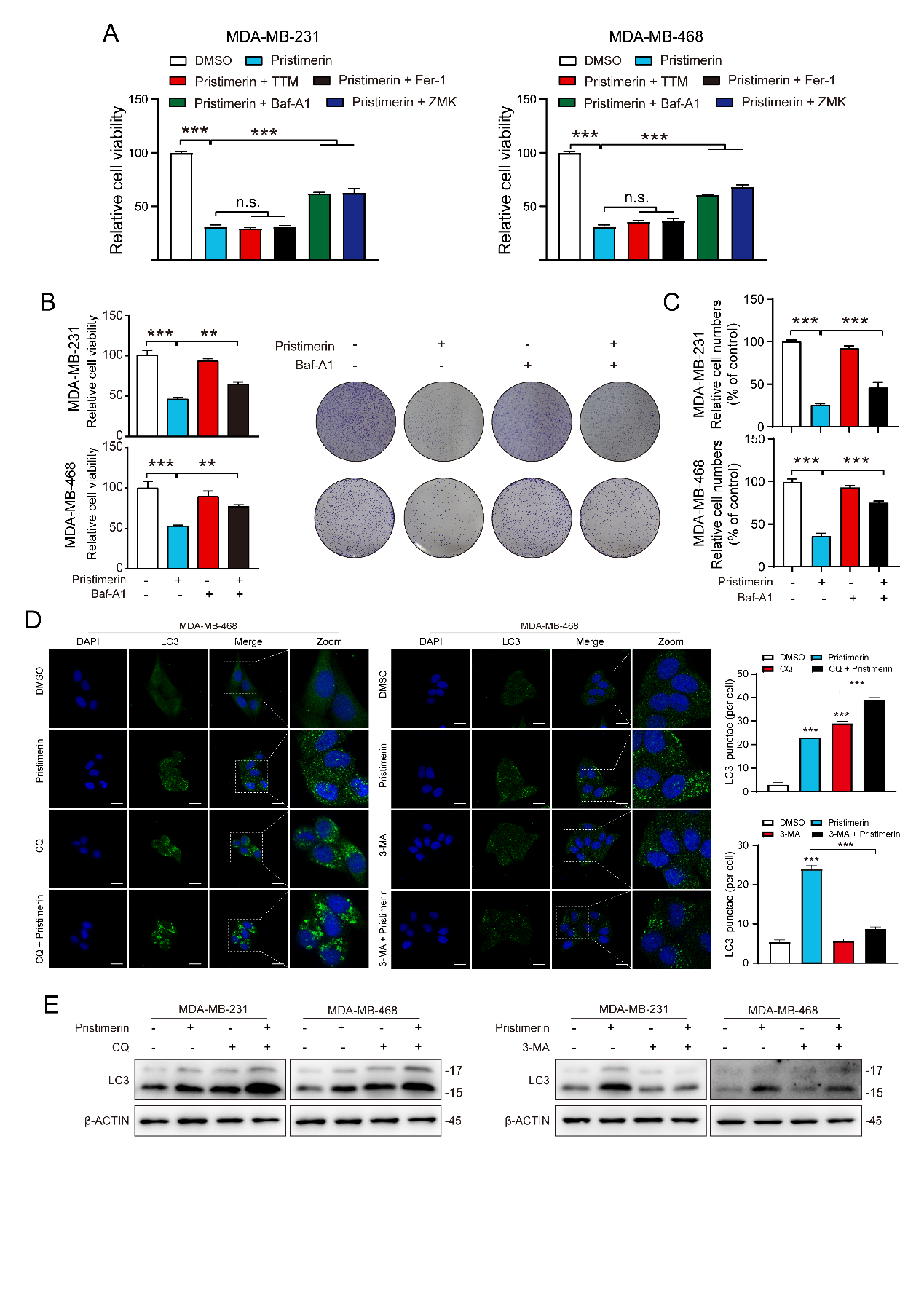


**Supplementary Figure S2.** (A) Cells were treated with pristimerin, with or without pretreated with 5 μM TTM, 1 μM Fer-1, 0.5 nM Baf-A1, 10 μM ZMK for 12 h, and then the cell viability was assessed by CCK-8. (B-C) Cells were treated with pristimerin, with or without pretreated with 0.5 nM Baf-A1 for 12 h, and then the cell viability and colony formation ability were determined by CCK-8 assay (B) and colony formation assay (C). (D) MDA-MB-468 cells were treated with pristimerin, with or without pretreated with 20 μM CQ or 1 mM 3-MA for 12 h, and then the LC3 puncta were examined by immunofluorescence. Scale bar = 20 μm. (E) Cells were treated with pristimerin, with or without pretreated with 20 μM CQ or 1 mM 3-MA for 12 h, and then the expression of LC3 was detected by Western blot. Bars, SDs; * 0.01 < *P* < 0.05, ** 0.001 < *P* < 0.01, and *** *P* < 0.001.

**
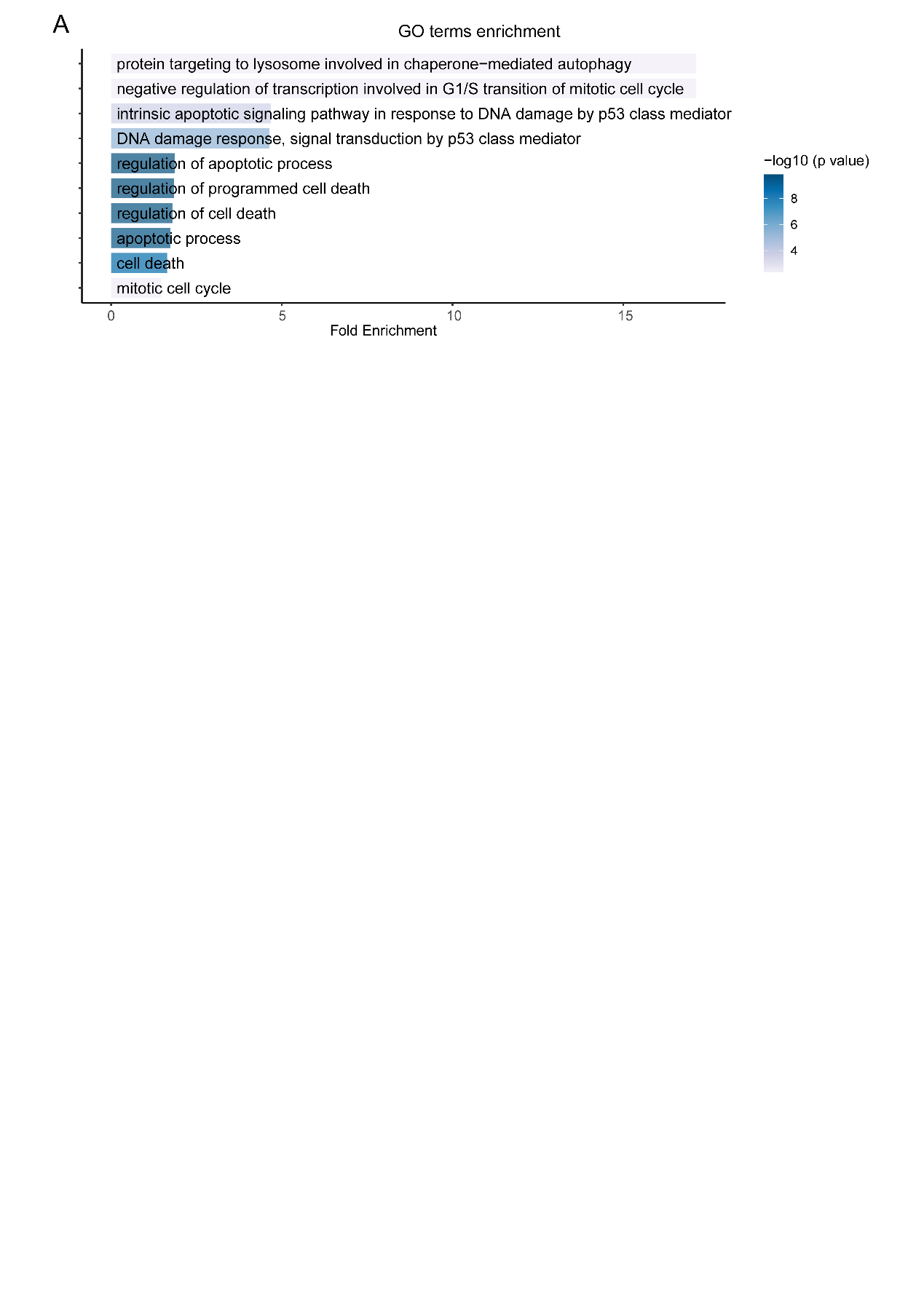
**

**Supplementary Figure S3.** (A) GO analysis reflected an enrichment of processes concerning cell autophagy, cell cycle arrest, cell apoptosis, and cell proliferation.

**
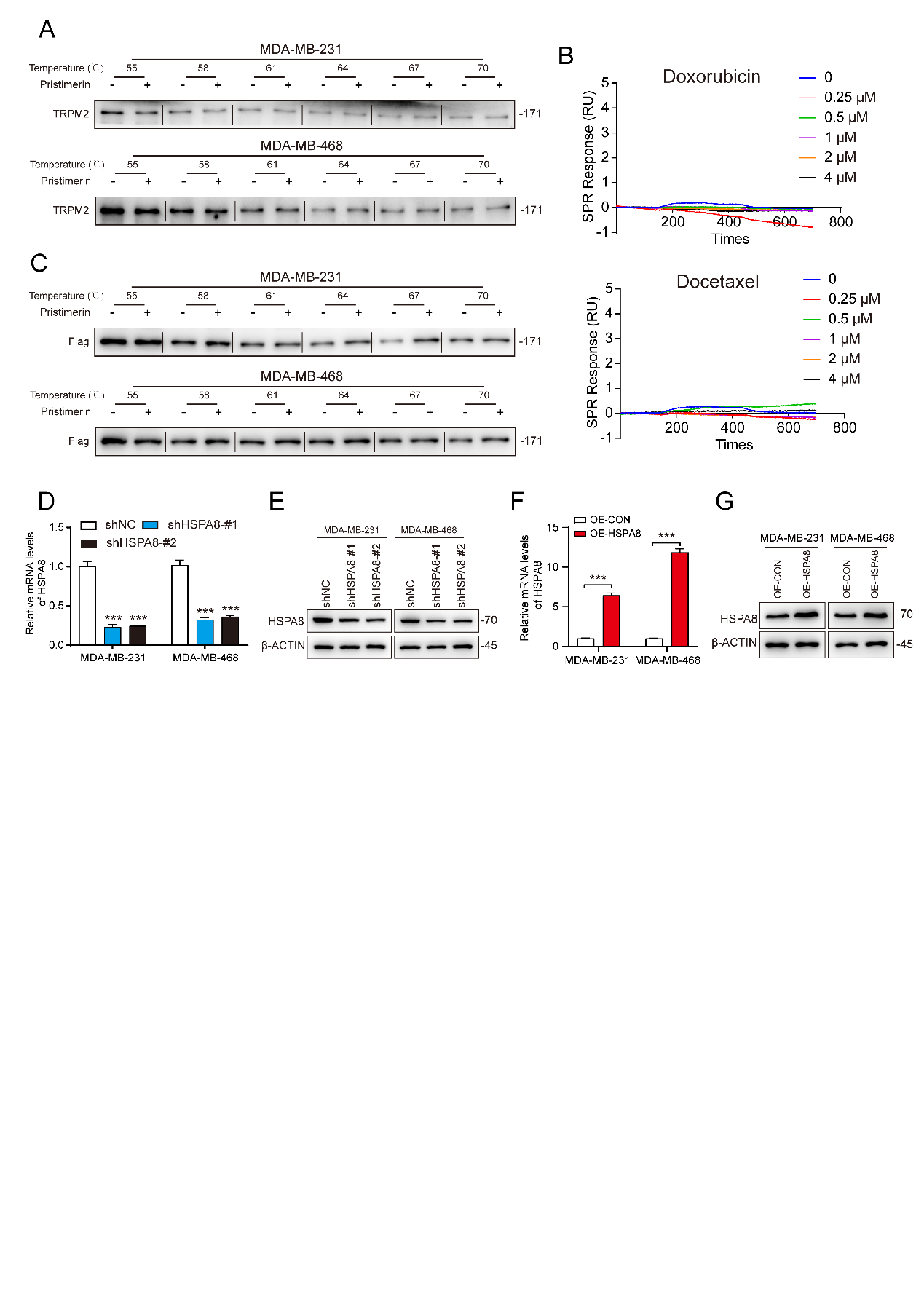
**

**Supplementary Figure S4.** (A) Western blot of CETSA for TRPM2 with pristimerin or DMSO for 1 h in MDA-MB-231 and MDA-MB-468 cells. (B) The binding affinity of HSPA8 recombinant protein with doxorubicin or doxorubicin were detected by SPR analysis. (C) Western blot of CETSA for HSPA8^S432A^-Flag mutants with pristimerin or DMSO for 1 h in MDA-MB-231 and MDA-MB-468 cells. (D) qPCR and Western blot were used to detect the expression of HSPA8 after transfection of HSPA8 knockdown plasmids and HSPA8 overexpression plasmid or vehicle. Bars, SDs; * 0.01 < *P* < 0.05, ** 0.001 < *P* < 0.01, and *** *P* < 0.001. n.s., no significance.

**
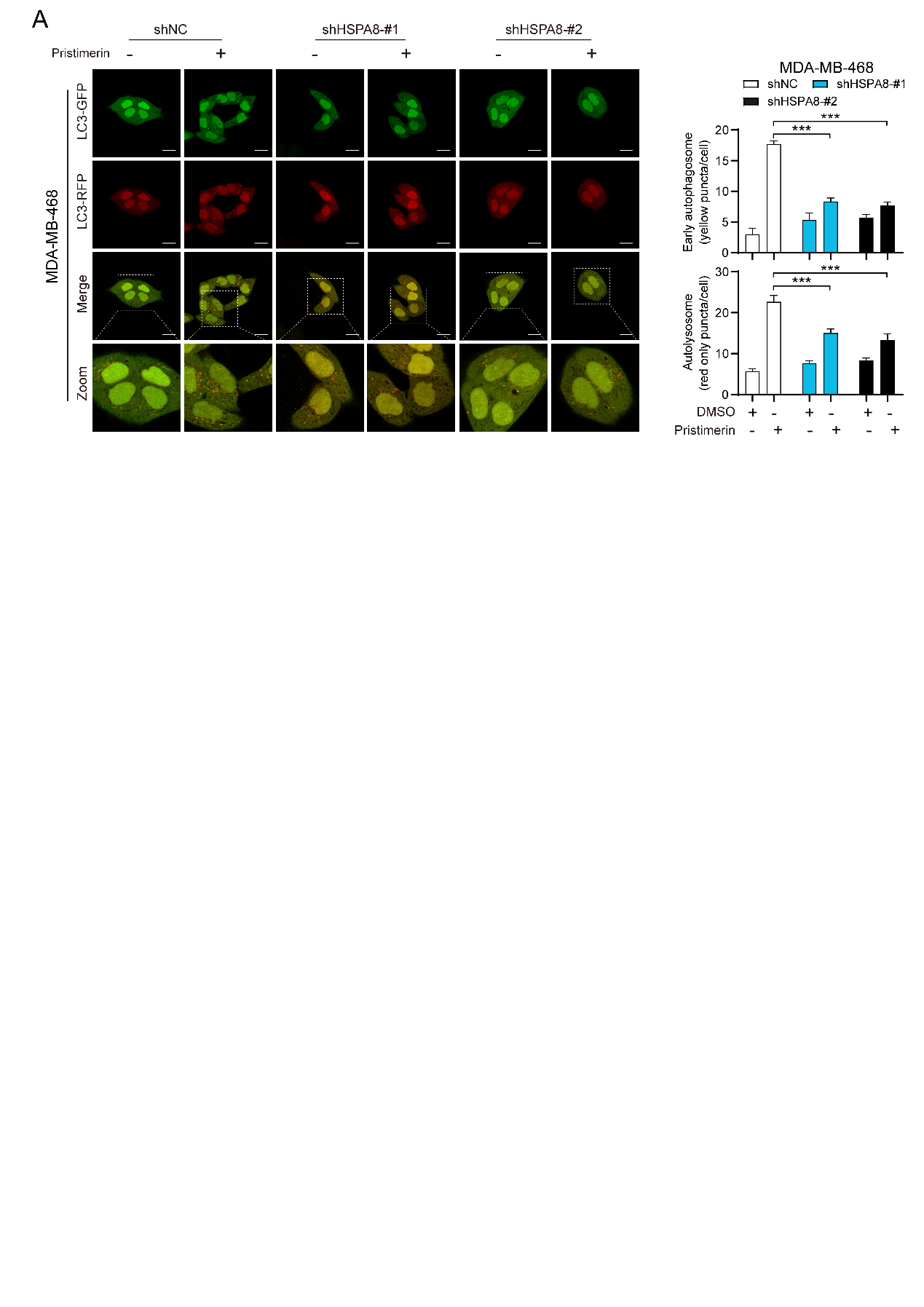
**

**Supplementary Figure S5.** (A) HSPA8 knockdown cells were treated with DMSO or pristimerin for 48 h, and then the autophagosomes and autolysosomes were examined by immunofluorescence. Bars, SDs; * 0.01 < *P* < 0.05, ** 0.001 < *P* < 0.01, and *** *P* < 0.001.

**
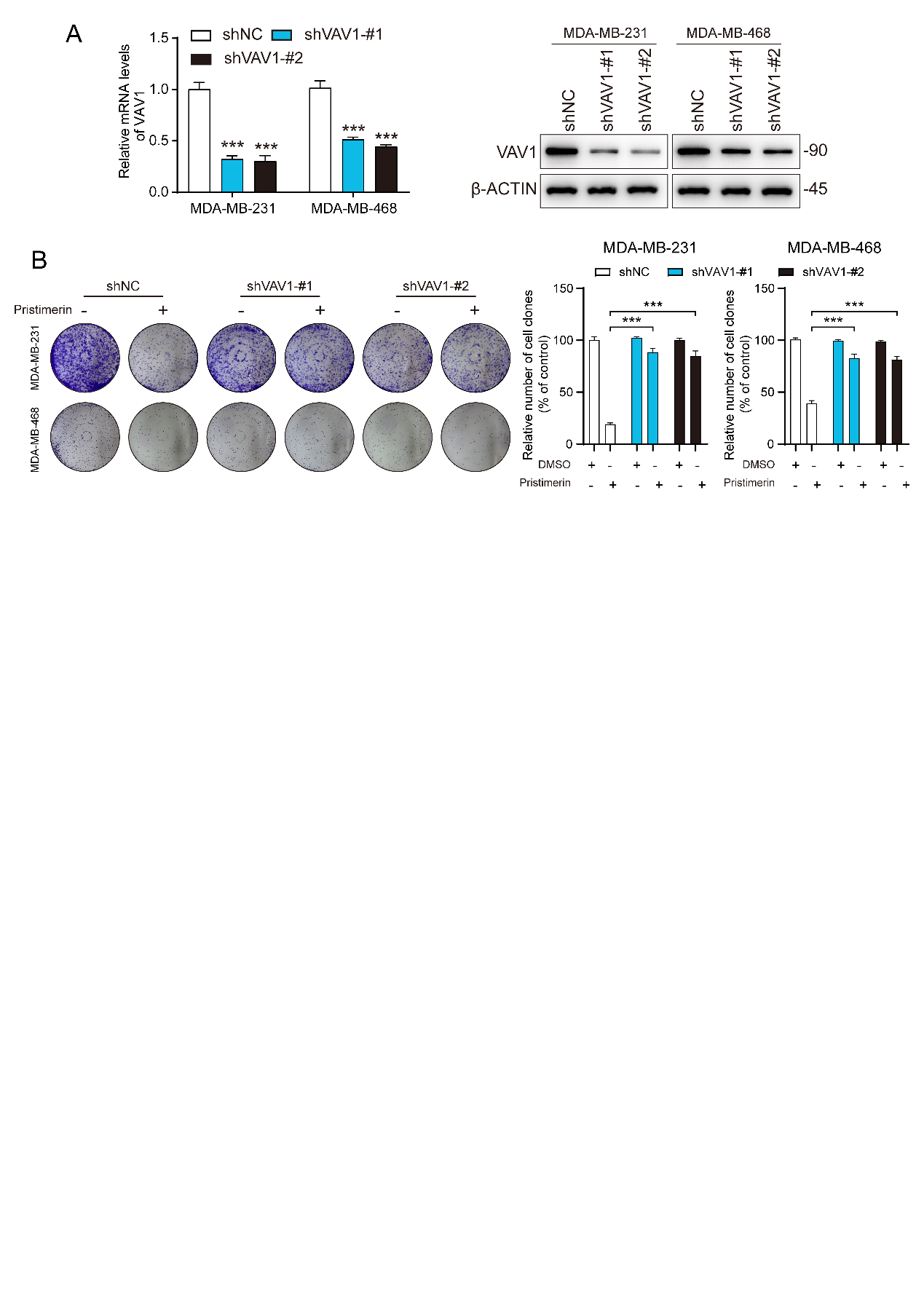
**

**Supplementary Figure S6.** (A) qPCR and Western blot were used to detect the expression of VAV1 after transfection of VAV1 knockdown plasmids or vehicle.

(B) The colony formation ability was detected in VAV1 knockdown cells combined with pristimerin treatment. Bars, SDs; * 0.01 < *P* < 0.05, ** 0.001 < *P* < 0.01, and *** *P* < 0.001.

**
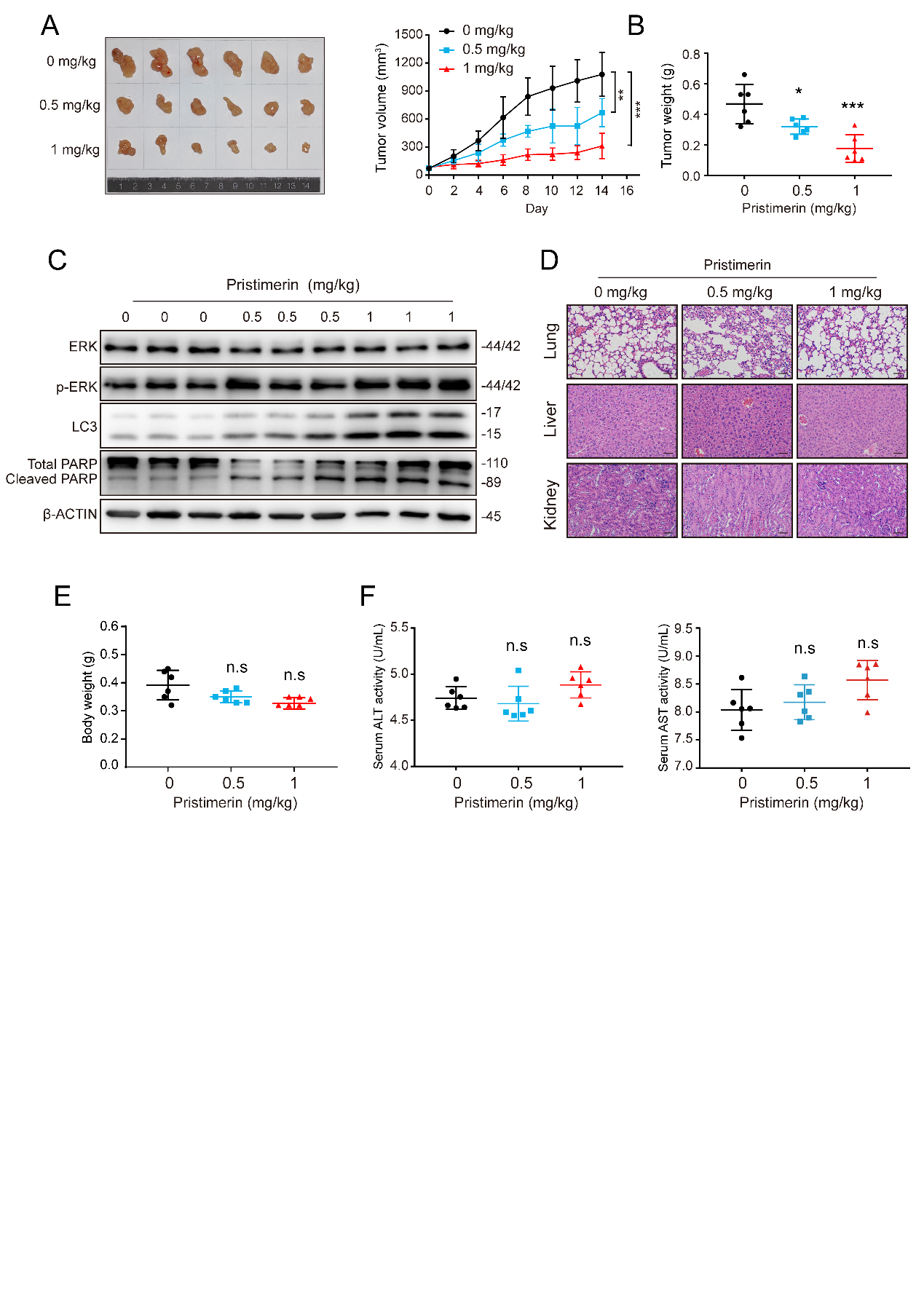
**

**Supplementary Figure S7.** (A-B) MDA-MB-468 cell was subcutaneously injected into the flanks of nude mice and palpable tumors were allowed to develop for 7 days. Mice were randomly divided into three groups (n=6) and treated with vehicle control (0.5% CMC-Na), 0.5 mg/kg pristimerin or 1 mg/kg pristimerin every other day. The tumor size was measured at indicated time intervals and calculated; at the end of experiment, tumors were excised, imaged (A) and weighed (B). (C) The expression levels of ERK, p-ERK, LC3 and Cleaved PARP in the tumors from mice treated with pristimerin or vehicle were detected by Western blot. (D) Hematoxylin and eosin (H&E) staining of lung, liver, and kidney specimens. Scale bar = 50 μm. (E) The body weights of nude mice at the end of experiment (n=6). (F) The serum levels of ALT and AST in nude mice at the end of experiment (n=6). Bars, SDs; * 0.01 < *P* < 0.05, ** 0.001 < *P* < 0.01, and *** *P* < 0.001. n.s., no significance.
